# Three-Dimensionally Printed Scaffolds and Drug Delivery Systems in Treatment of Osteoporosis

**DOI:** 10.3390/biomimetics10070429

**Published:** 2025-07-01

**Authors:** Cosmin Iulian Codrea, Victor Fruth

**Affiliations:** Department of Oxide Compounds and Materials Science, Institute of Physical Chemistry “Ilie Murgulescu” of the Romanian Academy, 060021 Bucharest, Romania

**Keywords:** osteoporosis, scaffolds, drug delivery systems, nanoparticles, biomimetic

## Abstract

The increasing incidence of osteoporotic fractures determines ongoing research on new methods and strategies for improving the difficult healing process of this type of fracture. Osteoporotic patients suffer from the intense side effects of accustomed drug treatment and its systemic distribution in the body. To overcome these drawbacks, besides searching for new drugs, 3D-printed scaffolds and drug delivery systems have started to be increasingly seen as the main strategy employed against osteoporosis. Three-dimensionally printed scaffolds can be tailored in intricate designs and make use of nanoscale topographical and biochemical cues able to enhance bone tissue regeneration. Research regarding drug delivery systems is exploring bold new ways of targeting bone tissue, making use of designs involving nanoparticles and intricate encapsulation and support methods. The local administration of treatment with the help of a scaffold-based drug delivery system looks like the best option through its use of the advantages of both structures. Biomimetic systems are considered the future norm in the field, while stimuli-responsiveness opens the door for the next level of efficiency, patient compliance, and a drastic reduction in side effects. The successful approval of these products still requires numerous challenges throughout the development and regulatory processes, but the interest and effort in this direction are high. This review explored various strategies for managing osteoporosis, emphasizing the use of scaffolds for targeted drug delivery to bone tissue. Instead of covering the whole subject, we focused on the most important aspects, with the intention to provide an up-to-date and useful introduction to the management of osteoporosis.

## 1. Introduction

Osteoporosis (OP) is a systemic skeletal chronic condition characterized by decreased bone mass and quality (microarchitectural changes) [1]. OP negatively influences bone regeneration potential, bone mineral density (BMD), and the ability to obtain proper mechanical stability for fracture healing [2]. As life expectancy increases, osteoporotic elderly patients, which represent a larger portion of the population, have a higher incidence of fractures not determined by significant trauma [1,3]. Also, there is an increasing incidence of osteoporotic fractures at all ages [4]. Statistical data indicate that approximately one-third of women and one-fifth of men worldwide either have or will develop OP [5]. Because such fractures usually do not heal, their healing time increases, or they heal inadequately, there is a high risk of subsequent fractures [6,7] and hundreds of billions of USD annually worldwide are spent for repair and regeneration [8]. Although both the public and academia have a high interest in the disease’s treatment [9,10], it is important to note that OP most often remains undiagnosed until an osteoporotic fracture occurs [11]. In the domain of medical imaging, central dual-energy X-ray absorptiometry (DXA) has been recognized by the World Health Organization as the gold standard for assessing BMD and diagnosing OP in postmenopausal women [12]. BMD represents a measure of both the quantity of bone tissue and its degree of mineralization, being influenced by the peak bone mass achieved during growth and the subsequent bone loss associated with aging, and is expressed as grams of mineral per square centimeter of scanned bone (g/cm^2^) [13]. A normal BMD is defined by a T-score of −1.0 or higher. Conversely, a T-score between −1.0 and −2.5 indicates low bone mass, or osteopenia, whereas a T-score of −2.5 or lower is diagnostic of OP [12]. OP may accelerate after the implantation of osteosynthesis devices, joint prostheses, or dental implants, causing poorer bone–implant adhesion and an increased probability of bone fracture [14].

Bone tissue is being continuously remodeled by bone-forming osteoblasts and bone-resorbing osteoclasts in healthy individuals in order to repair micro-damage and adapt to mechanical and metabolic needs [4]. In the initial stage of remodeling, old bone is removed by the osteoclasts, activated by the osteocytes, and, in the following stage, osteoblasts fill the areas previously resorbed by the osteoclasts [14]. Osteoblasts maintain and form healthy bone tissue through protein synthesis and matrix secretion [15]. New bone tissue comprise mostly collagen osteoid that is progressively mineralized [14]. Once an imbalance emerges between formation and resorption, mass loss begins, which leads to lower bone density compared to normal bone tissue. The excessive activation of osteoclasts and the increased osteoclast-induced bone turnover are the causes of this change in the normal equilibrium of the bone [6]. Important bone density loss usually affects the hips and vertebral bodies [16], and the common fragility fracture sites are the vertebral bodies, the proximal femur, the proximal humerus, wrist, forearm, and the distal radius [1,7]. OP-related fractures have significantly increased morbidity and have led to debilitating sequelae, like chronic pain, balance disorders [17], and premature mortality [18]. Although the majority of bone defects can heal naturally, the regeneration process may become challenging when OP leads to inadequate bone formation.

Primary OP, with distinguishable juvenile, postmenopausal, and senile forms, is the most frequent type. Secondary OP is derived from several other disease categories, such as endocrine (hypogonadism, hypocortisolism, hyperparathyroidism, acromegaly, diabetes mellitus), hematological, gastrointestinal, rheumatic, and kidney disease, or from the usage of drugs like glucocorticoids, anticoagulants, and diuretics [1,12]. The development of OP is influenced by multiple factors, including genetic predisposition, hormonal imbalances, the natural aging process, inadequate nutrition, and lifestyle habits such as smoking, reduced physical activity, or the usage of steroids [5,14]. In postmenopausal OP, the pathogenesis is associated with estrogen depletion, which enhances the bone loss that occurs with aging [1,18]. Estrogen depletion influences all bone cells by regulating cell differentiation and apoptosis and by changing the expression of estrogen response target genes, causing a faster increase in bone resorption [15].

Because the actual treatment of OP can cause significant side effects [5], research has focused on developing new safer drugs or drug formulations, on delivery methods of drugs at the defect site, avoiding as many side effects as possible, and on the development of 3D scaffolds that influence local bone tissue metabolism, promoting osteogenic induction. Conventionally, drug administration follows a systemic approach, where medications enter the bloodstream and circulate throughout the body. However, this method has several disadvantages, including potential toxicity, as well as insufficient penetration into the target tissue. Extensive damage can make traditional treatments ineffective, while bone grafting may be restricted due to the need for additional surgical procedures and the risk of disease transmission [19]. In such scenarios, bone tissue engineering has become a promising alternative, employing cells, scaffolds, and growth factors to restore damaged bone tissue, together with DDS. Various alternative treatment strategies for bone defects, such as distraction osteogenesis, growth factor delivery, electrical stimulation, the Masquelet-induced membrane technique, and their combinations have also emerged in recent years. Nonetheless, their clinical translation is hindered by prolonged treatment durations and a high risk of complications [20].

Growing interest in tissue engineering due to a growing demand for biodegradable biomaterials capable of promoting the repair, replacement, or restoration of hard tissues has resulted in the development of exciting potential alternatives to autogenous bone grafts [21]. Bone scaffolds designed for biomedical applications serve as three-dimensional (3D) frameworks that support bone regeneration and promote the formation of new bone tissue. It is important to note that personalized treatment could be considered depending on the physical conditions of the patient [18]. Bone tissue engineering not only refers to the use of a scaffolding material to induce the formation of bone from the surrounding tissue but also its acting as a carrier for implanted bone cells or other therapeutic agents [22]. Smart-material-based strategies encompass a variety of innovative applications, including smart scaffolds and stem cell constructs, intelligent drug delivery systems, and stimuli-responsive materials. These advanced biomaterials not only facilitate the targeted delivery of stem cells and enable the controlled spatial and temporal release of drugs and bioactive agents but also play a crucial role in biofilm modulation and infection prevention at wound sites [8]. Through the study of the characteristics and commonalities of diseases, the design of drug delivery systems (DDSs) has become a hot research topic at present. In the case of bone, with its limited blood supply, drugs administrated systemically are exposed to various physicochemical and biological factors that affect their bioavailability [23]. DDSs are designed to enhance the targeted delivery of specific therapeutic agents to desired sites more efficiently [24]. DDSs are especially useful when the drug has dose-limiting side effects, a narrow therapeutic window, and/or a short half-life that makes maintaining a proper drug concentration difficult [25]. Overall, nanostructured DDSs represent innovative alternatives for osteoporosis therapy by enabling controlled drug release, enhancing local drug concentration, reducing side effects, and promoting bone healing [26]. In this article, we have reviewed some of the most frequently employed anti-OP drugs; the main principles of bone scaffold development, as well as the most important biomaterials used for these scaffolds; and the main principles behind DDS development, as well as the most important DDS used in bone treatment. Finally, we have mentioned a few future perspectives regarding the strategies employed for fighting OP.

## 2. Osteoporosis Medication

The drug treatment of OP is considered an important research topic and important breakthroughs are expected. The existing OP treatments are mainly drug-based [4] and last long periods of time [7], and it seems that the drugs used have different anatomical efficacies [18]. There is a general research trend for optimizing and personalizing the treatment and reducing the side effects, as the general efficacy of antiresorptive drugs is still controversial, demanding more experimental and clinical studies [14]. A wide range of therapeutic agents have been introduced to the market; however, their clinical use remains significantly limited due to adverse side effects and high costs [27]. Considering the mode of action, drugs used in the therapy of OP fall in the general categories of antiresorptive or anabolic drugs. The ones used against postmenopausal OP include antiresorptives such as bisphosphonates (etidronate, clodronate, alendronate, risedronate, ibandronate, and zoledronate/zoledronic acid), which are the most used drugs for treating OP worldwide [28]; monoclonal antibodies like denosumab and romosozumab; selective estrogen receptor modulators (SERMs) like raloxifene and bazedoxifene; the anabolic teriparatide; and the dual-action, antiresorptive, and anabolic strontium ranelate [1,6]. Based on the source, mode of action, and characteristics of drugs, some authors have classified anti-OP therapy into the following categories: small molecular agents (bisphosphate, strontium salts, calcitriol or vitamin D, menaquinones or vitamin K, SERM, RANKL inhibitors, sclerostin inhibitors), monoclonal antibodies (anti-RANKL antibodies, anti-sclerostin antibodies), and synthetic peptides (calcitonin, PTH including teriparatide and abaloparatide, RANKL-binding peptide) [5].

Hormone replacement therapy, such as estrogen and estrogen–progestin, is effective for post-menopausal OP, but estrogen has poor tissue selectivity and severe side effects [27], causing an increased risk of coronary events, venous thromboembolism, stroke, and cancer [18]. Other less harmful options include supplemental calcium, sodium fluoride, vitamin D, and vitamin K [18].

Bisphosphonates (BPs), which are extensively used for OP treatment, act by inhibiting osteoclast resorption [16]. BPs, synthetic analogs of pyrophosphate, exhibit a strong affinity for hydroxyapatite (HA) on bone surfaces following absorption into the body. During bone resorption, osteoclasts internalize these compounds, which subsequently inhibit farnesyl pyrophosphate synthase activity, triggering osteoclast apoptosis. This process effectively reduces bone resorption while promoting an increase in bone density [28]. BPs raise concerns as their side effects are serious [16] and include osteonecrosis of the jaw (ONJ), atypical fractures [1], bone brittleness [14], and gastrointestinal disturbances [18].

Denosumab, a monoclonal antibody and RANKL inhibitor [5], acts by restraining the activity, survival, and recruitment of osteoblasts and seems to have the highest influence on the hip and femoral BMD of women with postmenopausal OP [29]. Denosumab is contraindicated in patients with hypocalcemia, as it further increases this problem, and skin infection is also cited as a side effect [1]. For men there are concerns due to a significant increase in cardiovascular events [17], and it is associated with more infections [28]. The antiresorptive denosumab increases bone density but also increases bone brittleness [14].

Romosozunab is a novel sclerostin antibody for the treatment of OP [29] with a dual effect of increasing bone formation and decreasing bone resorption, with limited adverse effects [18]. Romosozumab significantly enhances BMD and lowers the risk of fractures, especially when administered as a first-line treatment for patients at high risk of fractures. To maximize long-term efficacy, it is often followed by antiresorptive therapy in a sequential approach. Overall, it is well tolerated [30]. Nevertheless, a pharmacovigilance analysis based primarily on data from the US and Japan recommends a reduction in its use in osteoporotic patients with severe cardiovascular disease [31].

SERMs are synthetic compounds that bind to the receptor for estrogen. Raloxifene hydrochloride, a representative SERM, is used in the treatment and prevention of postmenopausal OP, but is related to adverse cardiovascular events, like pulmonary embolism and deep vein thrombosis, and even a higher risk of fatal strokes [32]. It is contraindicated in people with a history of venous thromboembolism [1]. Raloxifene exhibits poor water solubility and undergoes extensive first-pass metabolism in the liver, leading to an absolute bioavailability of approximately 2% [13]. By replicating estrogen’s protective role in bone health, raloxifene not only suppresses bone resorption but primarily stimulates bone formation. Its effects on bone tissue are mediated through estrogen receptors, activating pre-osteoblastic cells via the WNT/β-catenin signaling pathway. This pharmacological property makes raloxifene a compelling option for enhancing the functionality of bone grafts, implants, and composite scaffolds, as it can improve the osteogenic potential of biomaterials [33]. The development of novel crystalline and amorphous forms of raloxifene hydrochloride, alongside tailored delivery systems such as intravaginal rings and self-emulsifying tablets, exemplifies a targeted strategy for optimizing its therapeutic use in conditions like postmenopausal osteoporosis [32].

Teriparatide, the active fragment of parathyroid hormones (recombinant human PTHs 1–34), seems to be the most effective for preventing new hip fractures [18], but has contraindications regarding hypercalcemia, hyperparathyroidism, and renal impairment and, among other side effects, we can cite headache, nausea, dizziness, and postural hypotension [1]. Improvement in pain and functional ability is mentioned by patients using this treatment compared to the ones using BPs, but more extensive clinical evaluations, especially on long-term use, should be performed [34].

Strontium, usually as strontium ranelate, is effective at decreasing the risk of vertebral, nonvertebral, and hip fractures but is known for its link to thromboembolism and myocardial infarction in patients with cardiovascular risk [1,6]. It has a dual action by increasing new bone formation by osteoblasts and decreasing bone resorption by osteoclasts, and helps to increase the BMD in the hip and lumbar spine [18]. Strontium is the subject of ongoing research as new ways to bypass its systemic adverse effects are being investigated [35]. Besides the better known research regarding the integration of strontium into the HA matrix [36,37,38,39], new research proposes strontium–quercetin complexes [40], metal–phenolic networks, or strontium silicate particles [41].

Vitamin D can influence bone healing [2] and is recommended as an adjunct [7] in association with anti-osteoporotic drugs [17]. Vitamin D designates a cyclopentane polyphenanthrene group of compounds, with two primary forms, vitamin D_3_ and vitamin D_2_, derived from 7-dehydrocholesterol and ergosterol, respectively, both having structural similarities to cholesterol [42]. Vitamin D has long been regarded as essential for preserving bone health and both preventing and managing OP. However, in recent years, multiple observational studies and meta-analyses examining the effectiveness of vitamin D supplementation in reducing fracture risk among postmenopausal women have yielded inconclusive results [43]. Vitamin D is mentioned to enhance early-stage osteogenesis and angiogenesis while suppressing osteoclastogenesis when present in MgO-doped 3D-printed scaffolds [44] or quercetin containing encapsulated solid lipid nanoparticles [45]. Efforts undertaken to utilize vitamin D in different DDSs have demonstrated limited efficiency, especially concerning long-term storage [42]. Despite some conflicting findings, expert panels agree that ensuring a sufficient calcium intake and maintaining adequate vitamin D levels is essential before initiating anti-resorptive therapy in individuals with vitamin D deficiency. This recommendation is based on physiological reasoning, evidence from randomized controlled trials, and the minimal risks associated with supplementation. However, additional research, particularly from prospective studies, is necessary to determine the effectiveness of vitamin D when combined with anti-resorptive OP treatments [43].

Small interfering RNA (siRNA)-mediated gene silencing has more recently been applied to the treatment of bone diseases, including osteoporosis. The amount of research into this treatment has significantly grown because of its many benefits [29]. With this kind of treatment, siRNA was directed against the genes that had been found to suppress bone growth without altering bone resorption. To promote bone production significantly, siRNA must be administered at large concentrations, which increases the likelihood of negative effects on non-skeletal tissues [46]. Despite the therapeutic potential of siRNA-mediated gene silencing to enhance bone formation, its clinical application is hindered by challenges such as limited cellular targeting, rapid degradation, and poor membrane permeability. To overcome these barriers, nanocarriers, including liposomes, mesoporous silica nanoparticles, and cationic polymeric nanoparticles, have been employed to protect siRNA and enable its targeted delivery [47].

Statins, known as inhibitors of hydroxymethylglutaryl-CoA reductase and commonly used for cholesterol reduction, have also been shown to stimulate bone formation by upregulating the expression of bone morphogenetic protein-2 (BMP-2) in osteogenic cells. However, it is important to note that the administration of statins at high doses may be associated with an increased risk of developing osteoporosis [48].

These medications are typically administered systemically and, due to their limited bone-targeting efficiency and limited drug bioavailability, may require high doses and frequent dosing, resulting in severe side effects [23,48,49], while insufficient drug concentrations in bone diminish their therapeutic effectiveness. These challenges present significant obstacles for current anti-OP strategies (Table 1).

Among new anti-OP drugs, we can mention odanacatib, which is a selective and reversible inhibitor of cathepsin K, a tissue protease involved in bone resorption. Clinical studies have shown that odanacatib consistently increases BMD in the lumbar spine and hip while also reducing the risk of fractures. It significantly lowers biomarkers of bone resorption, such as serum type I collagen C-terminal peptides and urinary type I collagen N-terminal peptides. Additionally, transforming growth factor-beta (TGF-β) and growth differentiation factors (GDFs) are known inducers of osteogenesis and contribute to cellular differentiation and morphogenetic processes throughout the body. Platelet-derived growth factors (PDGFs) promote cell proliferation and migration, playing essential roles in embryonic development, tissue homeostasis, and bone formation. Activin A is a key regulator involved in RANKL-induced osteoclastogenesis. It functions through Smad-mediated signaling pathways, which are essential for the development and activity of osteoclasts. Activin A has emerged as a promising therapeutic candidate for the treatment of osteoporosis, particularly in cases associated with chronic kidney disease (CKD)-induced osteomalacia [51].

Due to the high cost of treatment, over 75% of osteoporotic patients in economically disadvantaged populations remain undiagnosed. Many women perceive the condition as a natural consequence of aging and endure its complications without seeking proper diagnosis. As a result, clinical data availability is severely limited. Addressing this issue requires collaborative efforts between researchers and healthcare professionals to advance research and develop more affordable therapeutic options [50]. This is highlighted by the fact that sequential drug treatment should be considered when bone resorption inhibitors fail or have been used for extended periods. Transitioning between different types of anti-osteoporotic drugs to maximize benefits and minimize adverse effects can add supplementary economic and compliance hurdles.

Traditionally, significant emphasis has been placed on the development and commercialization of new pharmaceutical compounds, while comparatively limited attention has been directed toward the advancement of DDSs aimed at optimizing existing drugs with identifiable shortcomings in their pharmacokinetic properties or routes of administration [25]. Many chemical and genetic drugs and even DDSs with great therapeutic potential may remain in the clinical research stage due to limitations such as poor solubility, poor stability, toxicity, impermeable biofilms, and poor targeting [52]. Therefore, the development of an advanced and efficient bone-targeting DDS is urgently needed for achieving the precise and effective treatment of osteoporosis [27,50].

## 3. Biomaterials in Osteoporosis Management

Tissue engineering aims to develop substitute tissues for regenerating and healing damaged tissue [53]. Bone defects associated with tumors, infections, or other bone pathologies present significant clinical challenges. While autologous and allogeneic grafts still remain the primary conventional treatments, their use is often limited by complications such as immune rejection, donor site morbidity, and infection risk [20]. Advance bone tissue engineering involves the use of scaffold structures to encourage new bone growth from adjacent tissues or to provide support for implanted bone cells and bioactive substances. These scaffolds, made from natural materials, synthetic ones, or their blends, offer benefits such as the ability to carry bone-forming cells and growth factors, thereby exhibiting both osteoconductive and osteoinductive properties essential for bone repair and regeneration, as well as biocompatibility, bioresorbability, and suitable mechanical strength [20,54]. The very foundation of bone tissue engineering rests on three essential components: scaffolds, cellular elements, and growth factors. Damaged tissue can be restored through two main strategies in tissue engineering: ex vivo and in situ approaches. In the ex vivo method, scaffolds are seeded with cells and biomolecules outside the human body to generate tissue constructs ready for transplantation. However, this approach presents several challenges, including tissue damage at the donor site, the necessity of obtaining large numbers of immunocompatible cells to colonize synthetic scaffolds, and difficulties associated with prolonged in vitro cell expansion under artificial conditions. These conditions often result in unreliable cell sources, inconsistent cellular phenotypes, and the inability to accurately mimic autocrine and paracrine signaling environments. Such limitations have led to increasing interest in in situ tissue engineering, which capitalizes on the body’s intrinsic ability to regenerate. This approach involves the use of biomaterials designed to deliver targeted biochemical and biophysical signals that stimulate the recruitment and activation of the body’s own cells at the injury site, promoting tissue repair and regeneration [55].

Bone grafting has evolved significantly, progressing from first-generation materials (metals and alloys like titanium, stainless steel, and Co–Cr alloys) to second-generation grafts (ceramics and polymers, including calcium phosphates, Al_2_O_3_, ZrO_2_, collagen, gelatin, chitosan, chitin, alginate, PLLA, and PLGA). The third generation introduced acellular composites or nanocomposites, while the fourth-generation incorporated composites or nanocomposites containing cells or cell-derived components. The anticipated fifth generation in bone grafting is centered around material design, leveraging additive manufacturing techniques alongside conventional methods to create optimal morphologies that enable deep cellular infiltration within the graft while also facilitating the controlled release of bioactive agents [6]. Conventional scaffolds often suffer from common drawbacks, such as infections linked to the materials used, structural weaknesses, and unfavorable immune responses from the host [8]. Materials like stainless steel, titanium, and chromium–cobalt alloys provide structural support for hard tissue replacement; however, these non-degradable materials have limitations in regard to adverse effects due to ion release, inflammation, or other biocompatibility issues [56].

To minimize the adverse effects associated with anti-OP drug administration, an effective approach would be the development of tissue-engineered scaffolds designed to influence local bone metabolism and promote osteogenic induction. Increasing evidence indicates that the micro- and nanotopographical features of scaffolds significantly impact osteoblastic cell function. Enhancing bone defect healing can be achieved by optimizing the surface architecture of bone grafts, creating a multiscale structural organization [57]. Designing multifunctional and adaptable cellular microenvironments is essential for creating biomaterials that mimic native tissues. The interface between cells and biomaterials forms a complex, dynamic microenvironment that plays a crucial role in tissue regeneration. When stem cells interact with scaffolds, they can detect various properties, such as stiffness and a nanostructure, and respond accordingly, allowing smart scaffolds to direct specific cellular behaviors. For instance, research has shown that innate immune cells, particularly macrophages, can undergo phenotypic shifts when exposed to tailored material cues, potentially enhancing the tissue regeneration process [8]. Biomaterials for in situ tissue regeneration have the ability to interact with and alter the in vivo microenvironment. This results in the need for regulatory approval for all aspects of safety and performance, including host–tissue receptivity, the effect on gene expression and signaling, short-term and long-term effects on the local microenvironment (including inflammation, foreign-body response, fibrosis, or rejection), and the safety or therapeutic effects of the degradation products. Accordingly, the additional verification and validation tests for biomaterials-based in situ regeneration require substantially more effort and resources than for bioinert scaffolds or devices. The in situ biomaterial systems available for clinical use include the INFUSE Bone Graft for orthopedic or dental applications [55].

Scaffolds can be categorized into two main types: non-degradable and biodegradable scaffolds. Non-degradable scaffolds are designed to remain permanently within the body, providing long-term structural support without undergoing degradation over time, while biodegradable scaffolds provide a mechanically resilient temporary framework that facilitates the migration and proliferation of new cells, playing a crucial role in tissue engineering and regenerative medicine applications [58]. Three-dimensional bioactive bone scaffolds can be fabricated from a wide range of biomaterials; however, bioceramics (e.g., tricalcium phosphate, biphasic calcium phosphate, bio-glasses) and biodegradable natural or synthetic polymers (e.g., collagen, fibrin, chitosan, polyesters, polydioxanone, polyethylene glycol) are particularly promising. Their composites offer an optimized approach for bone tissue engineering by combining the advantages of bioactive ceramics and biodegradable polymers [59]. With advances in imaging technologies such as CT and MRI, 3D models of a patient’s specific anatomy can now be created using 3D printing. Three-dimensional printing templates play a crucial role in orthopedic internal fixation surgery, particularly in the placement of pedicle screws during spinal procedures. Utilizing patient-specific 3D models can significantly simplify the process and enhance surgical accuracy [60]. Effective osteogenic scaffold design requires the biomimicry of native bone architecture through tunable synthetic or natural biomaterials. Advanced technologies like 3D bioprinting facilitate the fabrication of multiscale, multicellular constructs that replicate the structural complexity, functional diversity, and heterogeneous microenvironment of bone tissue [61].

The architectural design of scaffolds defines their macro- and mesoscale geometrical characteristics, which are critical for structural integrity and biological performance. The selected 3D printing technique significantly affects the scaffold’s micro- and nanoscale surface topography, influencing cellular interactions. Surface features can be further refined through post-processing treatments. Additionally, the chosen biomaterials contribute to subcellular-level geometry. Design strategies, such as Voronoi patterns, triply periodic minimal surfaces (TPMSs), tensegrity structures, raster-angle configurations, or auxetic geometries, play a pivotal role in determining the scaffold’s mechanical behavior and fluid transport properties, ultimately impacting its biological functionality and clinical effectiveness [62].

Calcium phosphate cements (CPCs) and biodegradable polymers are widely utilized as biomaterials for 3D scaffolds and in drug delivery systems due to their biocompatibility and degradability. Among these materials, aliphatic polyesters, proteins such as collagen and gelatin, and polysaccharides like alginate and chitosan can prolong drug release, ranging from several days to several months [6]. Collagen, which provides the interstitial framework for hydroxyapatite (HA) crystal deposition, enhances the mechanical properties of bone, including both tensile and compressive strength [60]. Also, HA has been explored as a carrier for growth factors, gene therapy vectors, and pharmaceutical agents [63]. HA-based nanoparticles can deliver both therapeutic agents and bone minerals directly to bone tissue. Beyond their drug delivery function, these nanocarriers actively contribute to bone regeneration by promoting bone mass deposition and stimulating the growth of new bone tissue [46]. HA is well known for its capacity to form direct bonds with living tissues, a characteristic largely attributed to its high affinity for type I collagen, positioning HA as an exceptionally promising material for promoting bone formation [48]. CPC NPs show high chemical and thermal stability, aptitude to either cation or anion doping, high adsorption capacity for organics (drugs and proteins), and pH-responsive solubility that opens the way to a controlled release of ions [64]. It is important to take into account anti-OP compounds that exhibit a high binding affinity to HA, which is the main component of bone [46]. NP incorporation into scaffolds has proven to improve the bulk mechanical strength of scaffolds [59]. Nanoscale HA demonstrates enhanced protein adsorption, improved cellular adhesion, and superior bioactivity compared to its microscale counterpart. It also significantly reduces apoptosis in healthy cells, thereby promoting cell proliferation and activity essential for bone regeneration [54]. CPCs can be 3D-printed into scaffolds with controllable nanopores and customized macropore structures [61].

The drawbacks of HA materials include a low degradation rate and relatively low drug-loading capacity, while achieving the usual production of uniform HA particles with consistent mono-dispersion and a narrow size distribution remains a significant challenge [65]. In the case of the degradation rate, better results can be obtained by ion substitution, a reduction in the crystallinity, and the introduction of calcium-deficient and amorphous phases [39,65]. Also, osteoinductive qualities are insufficient for allowing large bone defects to mend [54]. The wide variety of useful drugs, dosages, and functional groups frequently used to bind drugs to the CPC substrate opens up an almost unlimited number of variables in technologies used for the preparation of HA/drug compositions intended for targeted and controlled drug delivery [66].

Composite materials integrate the beneficial properties of multiple biomaterials; however, their processing must be carefully optimized to ensure that these combined advantages are effectively manifested. Additionally, it is essential that the fabrication techniques support adequate vascularization to promote successful tissue integration and regeneration [67]. Polymers modified with HA or other substances represent a novel strategy for enhancing the biocompatibility of nanomaterials [26] and more closely resemble the mechanical properties of bone [59]. The design of HA/polymer constructs for bone tissue engineering must address key structural and biological criteria. Structurally, these constructs should mimic native tissue in composition and architecture, provide suitable mechanical properties, and exhibit a highly porous, interconnected framework to support osteoconduction, osteoinduction, cell migration, vascularization, and nutrient exchange. Biologically, they must ensure biocompatibility, non-toxicity, non-immunogenicity, and controlled biodegradability to promote osteogenesis and successful host integration [54]. Nevertheless, HA-based composite formulations represent a promising strategy.

**Figure 1:**
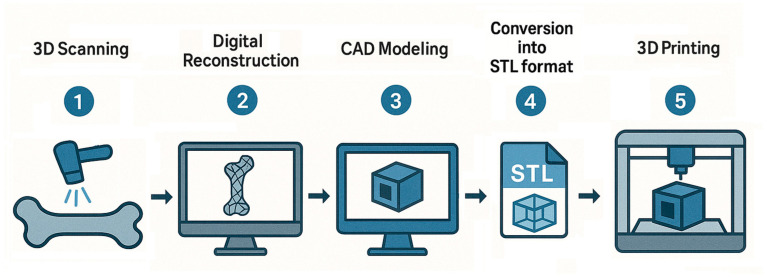
Three-dimensional printing process’s five key steps.

While bone tissue possesses an intrinsic self-healing capacity, critical-sized defects (typically > 2 cm) exceed this potential and require intervention. The limited availability of autologous grafts and donor-site morbidity underscore the need for biocompatible, bioactive bone substitute materials as viable clinical alternatives. Three-dimensional printing enables the fabrication of functional scaffolds that replicate the native tissue microenvironment by using appropriate biomaterials and cell types. It allows for precise control over scaffold geometry, porosity, and interconnectivity, promoting cellular infiltration and tissue regeneration [68]. When integrated with medical imaging data, such as computed tomography (CT) and magnetic resonance imaging (MRI), this approach streamlines the creation of patient-specific constructs precisely tailored to the affected anatomical regions. The 3D printing process typically involves five key steps: (i) acquisition of anatomical data through 3D scanning, (ii) reconstruction of the digital anatomical model, (iii) computer-aided design (CAD) modeling of the structure, (iv) conversion of the model into STL format, and (v) execution of the actual 3D printing process [69] (Figure 1).

Despite its great advances and potential, obviously not all 3D printing is well suited for orthopedic applications. Needless to say, there is no gold standard established yet from the vast array of techniques, and more research is needed to find it among the most commonly used techniques (Table 2).

The primary applications of 3D printing in orthopedics include medical education, orthotic fabrication, surgical planning, the development of surgical guides, and the production of patient-specific implants. Three-dimensional digital models, generated from tomographic imaging data, can be transformed into physical models that support preoperative planning. These models facilitate the design of customized surgical guides and implants, offering orthopedic surgeons enhanced precision and support in managing complex clinical cases [69].

Despite its clinical potential, the widespread adoption of 3D printing technology is limited by cost-effectiveness and implementation challenges. Ongoing expenses include materials, equipment maintenance, and specialized personnel training. However, potential cost savings may arise from a reduced operative time, lower revision rates, and improved surgical outcomes. For instance, 3D-printed surgical guides for complex acetabular fractures significantly decrease operative time and blood loss. Successful clinical integration requires adherence to regulatory standards, quality control, and streamlined workflows, including standardized protocols for image acquisition, segmentation, design approval, and print validation [72].

Three-dimensional printing enables the customization of scaffolds tailored for large bone defect regeneration. However, the intricate and dynamic nature of tissue regeneration, combined with patient heterogeneity, poses significant challenges for the systematic design of patient-specific scaffolds. To address these complexities, integrating enabling technologies such as in silico modeling, omics, bioinformatics, and information technology with 3D printing is essential. Systems biology approaches, including omics and bioinformatics, can distill complex biological data into actionable insights. These insights can then be incorporated into computational models that simulate bone regeneration processes, ultimately facilitating the optimization of scaffold design for precision and personalized therapeutic applications [62].

Key challenges hindering the application and widespread adoption of 3D printing in orthopedics include (i) the need for complex image preprocessing by trained personnel to generate usable digital models; (ii) a trade-off between implant strength and imaging compatibility, as high-density materials may hinder postoperative radiographic evaluation; (iii) the limited biocompatibility of printed constructs, necessitating the adoption of multimaterial printing strategies; (iv) the insufficient application of topology optimization to both surface and internal structures for enhanced cell adhesion and vascularization; and (v) the need for more extensive in vitro studies in tissue engineering to validate functional outcomes [73]. Despite the advantages of producing an exact geometry in biomedical devices, 3D-printed objects cannot yet mimic human tissues closely [74].

The integration of 3D printing technology in both preoperative planning and intraoperative procedures has significantly enhanced clinical outcomes. Reported benefits include improved surgical precision, a reduction in intraoperative blood loss, shortened operative and fluoroscopy times, and a decreased learning curve for complex procedures. Moreover, the use of patient-specific instruments can augment surgical safety and accuracy. Additionally, 3D-printed anatomical models facilitate clearer patient–physician communication by providing tangible visual aids for informed decision-making [75].

## 4. DDS for Osteoporosis Treatment

A DDS is designed to introduce therapeutic agents into the body while enhancing their safety and effectiveness by regulating the rate, timing, and location of drug release, as well as their bioavailability [66,76]. DDSs are usually composed of two main components: the actual therapeutic agents and the delivery vehicles or carriers necessary for transporting the therapeutic agents to their target sites. The therapeutic agents can be attached to the surfaces of the carriers or encapsulated within them. The carriers include liposomes, polymeric micelles, NPs, carbon nanotubes, dendrimers, and graphene oxide [24,77], while the therapeutic component can include a wide variety of agents, such as small molecules, cytokines, peptides, proteins, and genes [8]. Also, DDSs are based on diffusion, chemical reactions, solvent interactions, and stimulus-responsive mechanisms as the primary modes of drug release [78]. Given the significant variability in biological barriers and disease conditions among and within patient populations, it is essential to develop delivery methods that are highly adaptable and customizable to ensure effective therapeutic administration. To address the extensive variability in biological barriers and disease conditions both within and across patient populations, it is crucial to develop therapeutic delivery methods that are highly flexible and customizable. It is even suggested that each produced medicine requires the creation of unique delivery mechanisms, which adds a high level of complexity to the subject [46]. These delivery challenges are further compounded by patient comorbidities, different stages of disease progression, and individual physiological differences [79]. The well-established cytocompatibility and tissue integration of bioceramic NPs can be further improved by combining them with polymeric materials to develop bone-mimicking platforms for targeted treatment of bone pathologies [26]. However, these DDSs still present challenges, such as potential cytotoxicity, limited biodegradability, and possible adverse immune reactions, highlighting the need for further research to address these limitations [8].

Biomimetic materials are engineered to replicate specific features or functions observed in biological systems. In recent years, biomimetics has emerged as a key concept in the biomedical field, particularly in the design of DDSs. This approach offers a promising platform for achieving more sophisticated and efficient therapeutic delivery [76]. “Drawing from a gourd” biomimetic strategy involves replicating macro- or micro-scale structures found in animals and plants to enhance or optimize functional designs [52].

### 4.1. Route of Administration

**Figure 2:**
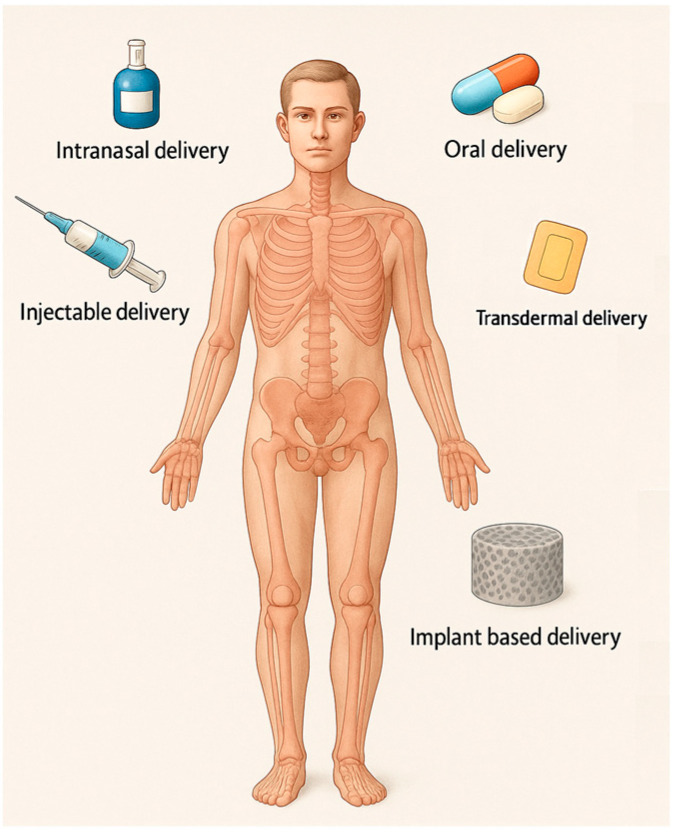
Route-of-administration DDSs used in anti-OP treatment.

The FDA approved several DDSs grouped by route of administration: oral, intramuscular, transdermal, subcutaneous, intraocular, intranasal, intrauterine, and transmucosal (pulmonary, sublingual, buccal, intravaginal, and rectal) [25], but not all of them are suited for bone treatment. DDSs used in anti-OP treatment (Figure 2) can be categorized based on their route of administration as follows:

1. Oral administration DDSs. Oral administration is broadly considered the safest, most convenient, and economically advantageous route for drug delivery. It is commonly used but has limitations such as low bioavailability and adverse effects due to systemic distribution. Although oral drug delivery is highly desirable from a patient care perspective, it faces substantial physiological challenges that compromise drug stability, absorption, and overall bioavailability. The acidic gastric environment promotes acid-catalyzed degradation, resulting in the loss of drug efficacy. In addition, enzymatic degradation, primarily through proteolytic enzymes, leads to the breakdown of macromolecular therapeutics and other labile compounds prior to their absorption. The gastrointestinal tract further presents physical and biochemical barriers, including the mucus layer and the epithelial lining. The mucus entraps larger molecules, impeding their diffusion, while the tight junctions of the epithelial cells limit paracellular transport, thereby restricting the systemic uptake of poorly permeable drugs. Moreover, orally administered drugs are subject to first-pass hepatic metabolism, wherein a considerable fraction is metabolized by liver enzymes before reaching systemic circulation, significantly reducing therapeutic availability [80].
2. Parenteral Administration DDSs. Injections can improve bioavailability but still pose challenges like patient compliance and side effects. When recombinant salmon calcitonin is utilized in the form of injections, it is associated with adverse effects such as nausea and facial rash, attributable to the high plasma concentration peaks following administration [13]. DDSs that avoid first-pass metabolism by using a parenteral route to deliver a drug directly into the bloodstream enable comparatively less material to achieve the same therapeutic effect in a well-controlled manner [25].
3. Transdermal patch and microneedle DDSs. These systems, such as those developed for risedronate, are used as an adverse-effects-reducing method, with the potential to improve patient compliance [81]. Transdermal microneedle array patches deliver drugs to the epidermis or upper dermis, avoiding the cutaneous pain receptors and allowing for painless drug delivery [25]. The avoidance of drug degradation within the gastrointestinal tract, coupled with the circumvention of hepatic first-pass metabolism, enables controlled drug delivery, enhances tolerability, and broadens the potential scope of therapeutic applications. For a drug to successfully reach systemic circulation via transdermal delivery, it must traverse the aqueous environment of the viable epidermis. Molecules exhibiting a Log P (octanol–water partition coefficient) value between 2 and 3, indicative of intermediate lipophilicity, are considered optimal candidates. Additional requirements include a molecular weight below 500 Da, sufficient solubility, low ionization, high pharmacological potency, and good skin tolerability [13].
4. Intranasal administration DDSs. For drugs like raloxifene, intranasal delivery using chitosan nanoparticles can enhance bioavailability and provide a promising alternative to oral administration [82]. Challenges come from the fact that the nasal mucosa contains a diverse array of xenobiotic-metabolizing enzymes, including cytochrome P450-dependent enzymes, as well as those involved in phase I and phase II metabolic pathways. Additionally, the nasal mucociliary clearance system constitutes a critical component of the nasal cavity’s innate defense mechanisms, playing a key role in protecting against inhaled pathogens and foreign substances [83]. Bisphosphates and hormones have been used to treat osteoporosis, with treatment approaches like oral and intranasal delivery [48]. Recombinant salmon calcitonin administered via nasal spray can lead to complications, including epistaxis, rhinitis, and ulceration of the nasal mucosa [13]. This route is used more often to deliver anti-seizure medications, migraine medications, cholinesterase inhibitors, and antidepressants [25].
5. Local DDSs offer a promising strategy for osteoporosis treatment by targeting therapeutic agents directly to bone tissue. Approaches such as bone-targeting nanoparticles and hydrogel implants enhance drug localization, reduce systemic side effects, and improve efficacy. While encouraging results have been observed in preclinical studies, these technologies remain largely experimental. As drug-loaded bone scaffolds function as localized DDSs, they are capable of targeting bone tissue locally and specifically, thereby facilitating the further treatment of the injured area and promoting the healing process. Bone scaffolds can function as prolonged-release platforms for therapeutic agents. This can be achieved either by direct drug incorporation into the scaffold or by embedding drug-loaded nanoparticles, which address issues such as toxicity and poor solubility inherent to many drugs. Additionally, adjuvant therapies can enable precise cell targeting through strategies such as stimulus-responsive nanocarriers or the surface functionalization of carriers with targeting ligands [59]. Some authors mention that DDSs in preclinical development for OP include HA-containing scaffolds [25]. This is further discussed in the following section, titled 3D scaffold-based systems.

### 4.2. Compositional and Structural Types

The physicochemical properties of a drug are determined by its components, which in turn influence how it interacts with the body and the physiological changes that it induces. Each DDS possesses unique characteristics that govern its release rate and mechanism, primarily influenced by its physical, chemical, and morphological properties, which in turn affect its affinity for different drug substances [78].

A wide array of specialized drug delivery systems, including aquasomes, pharmacosomes, dendrimers, bilosomes, virosomes, cubosomes, emulsomes, transferosomes, and resealed erythrocytes, have been developed to ensure the precise and efficient transport of therapeutic agents within the body. A recent review cites the following types of innovative DDSs for different therapeutic purposes: red blood cell membrane-camouflaged nanoparticles, hyaluronic-acid-based drug nanocarriers, hexagonal boron nitride nanosheets, polymer–lipid hybrid nanoparticles, self-microemulsifying, in situ gel, and micro-electromechanical systems [78].

Aquasomes are nanocarriers capable of preserving the structural integrity of bioactive molecules while enabling their controlled release at target sites. Pharmacosomes, formed through the conjugation of drugs with lipids, facilitate targeted delivery and improved pharmacological performance. Dendrimers, with their highly branched and tunable architecture, provide versatile platforms for drug encapsulation and sustained release. Bilosomes, composed of liposomal structures integrated with bile salts, enhance the ability to traverse biological barriers. Virosomes emulate the mechanisms of viral entry to achieve efficient intracellular drug delivery. Cubosomes, characterized by their unique cubic liquid crystalline structure, offer high stability and a sustained release profile. Emulsomes, lipid-based nanocarriers, are particularly effective for the encapsulation and delivery of poorly soluble drugs. Transferosomes, deformable vesicles, enable transdermal drug delivery by navigating through narrow intercellular spaces. Lastly, resealed erythrocytes, engineered from autologous red blood cells, serve as biocompatible carriers with prolonged systemic circulation [84].

DDSs used in anti-OP treatment can be categorized based on their structure as follows (recent examples in Table 3):

1. Nanoparticle-based systems. A promising approach for DDSs involves leveraging innovative nanoparticles (NPs) while using conventional medications. NPs (1–100 nm size range) have been extensively researched for their unique physical and chemical characteristics, making them valuable in biomedical applications such as disease diagnosis and treatment, biological imaging, and biosensor development [24]. Also, nanotechnology has been applied in orthopedics for bone tissue engineering, implant and prosthesis surface modification, targeted drug delivery, and advanced diagnostics [26]. Among their useful proprieties are their small size, large surface area-to-volume ratio, and tunable surface characteristics [48]. The pharmacokinetics of nanoparticle-based DDSs are primarily influenced by their physicochemical characteristics, which are determined by factors such as material composition, size, shape, surface charge, and modifications. NPs can enhance the stability and solubility of encapsulated compounds, facilitate membrane penetration, and extend circulation time, ultimately improving both safety and therapeutic effectiveness [79] while reducing formulation costs [76]. NPs offer several advantages, including a high drug-loading capacity relative to their size, enhanced solubility, improved drug stability, reduced side effects, and increased efficiency in facilitating drug transport and internalization within specific organelles [48]. The diverse range of requirements can be met by designing NPs tailored to specific patient groups, disease types, or a combination of both [79]. Numerous foundational studies have explored material selection and size optimization to enhance DDS effectiveness. Encapsulating conventional drugs within nanoparticles has been shown to enhance targeting precision while minimizing adverse effects in the treatment of various diseases [24]. The following classification of NPs used for DDSs was made by some authors: inorganic nanomaterials (silica NPs, HA NPs, metallic NPs, magnetic NPs, titanium nanotubes, nanospheres of mesoporous bioactive glass, etc.), polymeric NPs (gelatin NPs, chitosan NPs, nanogels, polyurethane nanomicelles), and lipid-based NPs [46,48]. Inorganic NPs, encompassing metals, metal/non-metal oxides, semiconductor NPs, etc., exhibit unique properties, such as surface effects, quantum size effects, and macroscopic quantum tunneling effects, while organic NPs, such as polymeric micelles, vesicles, liposomes, and dendritic polymers, hold a central role in improving the stability of drugs and genes. Nanoparticles composed of calcium phosphate have demonstrated the ability to activate cellular functions, enhance mineralization processes, and stimulate bone tissue growth [48]. Bioceramic NPs are the best alternative for reparative and restorative bone treatment due to their biomimetic composition, bioactivity, and good assimilation into the natural bone structure [26]. Organic nanocarriers enhance solubility, refine pharmacokinetic profiles, promote accumulation at targeted sites, and contribute to the reduction in systemic toxicity and adverse effects, thereby optimizing therapeutic efficacy [76]. It is worth mentioning also that microparticles, typically ranging from 1 to 1000 μm in size, are also studied. Microparticles are particularly effective for the delivery of larger therapeutic payloads and facilitate localized, controlled drug release at the target site [80]. NPs face limitations in regard to their potential antigenicity and immunogenicity, interacting with the mononuclear phagocyte system or the complement system, which leads to their rapid clearance [76].
2. Liposome-based systems. Liposomes have become one of the most prominent nanocarriers in the field of targeted drug delivery, owing to their low immunogenicity, high degree of versatility, and well-established therapeutic efficacy. A liposome is a closed, spherical, lipid bilayer artificial membrane, similar to a cell membrane, that forms an internal cavity capable of carrying various substances [85]. However, the physiological characteristics associated with each specific disease vary considerably, necessitating the tailored formulation of each liposomal system to align with the particular pathological conditions [86]. Used for bone-targeted delivery, liposomes can encapsulate drugs and deliver them specifically to bone tissue, although clinical translation remains challenging [87]. An example of a bone-targeting liposome is the system containing an oligopeptide of eight aspartate residues (Asp8), which had previously been shown to specifically target the bone, encapsulating icaritin. In vivo, researchers found that the Asp8-icaritin-liposome enhanced bone formation in ovariectomized mice compared to an icaritin-liposome control lacking the Asp8 moiety [88]. Other authors mention developing an α-cyperone-containing liposome-based nano-drug delivery system that specifically targets the bone resorption interface [89]. Liposomes can be tailored to be pH- or temperature-sensitive. Concerns exist regarding the long-term stability of liposomal formulations. Due to their high surface-area-to-volume ratio, liposomes possess elevated surface free energy, which can result in physical instabilities such as particle aggregation or fusion. These phenomena may lead to an increased particle size and unintended drug leakage. In addition, chemical instabilities, such as the degradation of ligands conjugated to the liposomal surface, can adversely affect the precision and efficacy of the targeting mechanism [86].
3. Solid lipid nanoparticle-based systems. Solid lipid nanoparticles (SLNs) are potentially biocompatible and efficient nanocomposite systems. SLNs were developed as an alternative to liposomes. They are colloidal dispersions in an aqueous medium, characterized by a matrix composed of solid, biodegradable lipids [90]. These systems offer several significant advantages, including sustained drug release, enhanced bioavailability, improved drug encapsulation efficiency, and broad applicability across various therapeutic areas [91,92], and facilitate the use of poorly water-soluble active pharmaceutical ingredients [93]. When they are combined with thermosensitive injectable sol–gel systems, SLNs turn into an effective and efficient drug delivery system [94]. SLNs can incorporate both hydrophilic and lipophilic compounds within their matrix [91]. Biocompatible lipids such as Compritol^®^888 ATO, Precirol^®^ ATO5, cetyl alcohol, cetylpalmitate, glyceryl monostearate, trimyristin/Dynasan^®^114, tristearin/Dynasan^®^118, stearic acid, and Imwitor^®^900 are used in the formulation of SLNs and appear to be well tolerated physiologically when administered in vivo [49]. Nanostructured lipid carriers (NLCs), representing the next generation of SLN-based systems, are composed of solid and liquid lipids at the nanoscale and have been developed to address certain limitations associated with SLNs, including their limited drug-loading capacity and the tendency for drug expulsion during storage [49,90]. Future developments of SLN anti-OP solutions will focus on the development of reproducible and scalable formulations of a lipid carrier for poorly soluble drug substances.
4. Hydrogel-based systems. Hydrogels are 3D networks composed of hydrophilic polymers that, through the chemical or physical cross-linking of polymer chains, can retain substantial amounts of water while preserving structural integrity. This architecture enables the encapsulation of therapeutic agents, including nucleic-acid-based drugs, thereby forming a sustained-release reservoir. The release kinetics can be modulated by adjusting the degree of cross-linking within the hydrogel matrix [80]. Injectable hydrogels represent a highly promising approach for localized DDSs in the management of bone-related conditions such as osteoporosis, osteonecrosis, osteoarthritis, osteomyelitis, and osteosarcoma, offering a viable approach for mimicking the topography and properties of the extracellular matrix [95]. The development of advanced smart hydrogels and nanogels that respond dynamically to external stimuli, such as changes in pH or temperature, offers a promising strategy for improving controlled drug release. These materials can undergo reversible structural transformations, thereby enhancing delivery precision and therapeutic efficacy. Furthermore, integrating hydrogels with nanogels may combine their respective advantages. For example, the use of biopolymers can enhance structural stability, mechanical strength, and drug-loading capacity. Incorporating nanoparticles into hydrogel matrices may further improve both mechanical performance and the efficiency of drug encapsulation [96]. Key challenges include ensuring mechanical stability and controlled degradation, achieving precise drug release and optimal bioavailability, and managing immune responses and ensuring biocompatibility, as well as addressing issues related to scalability and reproducibility in production [95]. Polymers can act as carriers that increase drug efficacy and reduce adverse effects by allowing for localized and prolonged drug release. They improve patient compliance by reducing the frequency of drug administration [23]. The ability to release biomolecules at a desired rate will play critical roles in the future development of bone scaffolds [22].
5. Moiety-based systems. These systems use water-soluble polymers to direct drugs specifically to some tissues, reducing side effects and improving therapeutic efficacy. Surface functionalization for targeted drug delivery represents a promising approach for reducing systemic toxicity and off-target effects commonly associated with nonspecific drug distribution. By attaching targeting moieties to the surface of drug carriers, the specificity of delivery to diseased tissues can be significantly enhanced. In particular, ligand-mediated strategies, employing antibodies, peptides, or small molecules that selectively bind to overexpressed receptors on target cells can substantially improve the accuracy and therapeutic efficiency of the delivery system [96]. Other authors mention as well that, by coating the inorganic NP with additional surface ligands (i.e., proteins, peptides, carbohydrates, etc.), higher reactivity and enhanced functionality can be achieved [54].
6. Stimuli-responsive systems. Researchers are exploring strategies to regulate the spatial and temporal release of therapeutic agents from controlled and targeted DDSs, such as on-demand release with stimuli-responsive functional groups after advancing to the targeted tissue [77]. This allows therapeutic agents to be precisely delivered to the intended site at the optimal dosage. Several types of stimuli-responsive smart materials have been developed for drug delivery. This strategy ensures a customized spatiotemporal release, reducing side effects while maximizing therapeutic impact. Stimuli-responsive biomaterials have the ability to react to external or internal triggers, including mechanical forces, electrical or magnetic fields, temperature fluctuations, pH variations, and enzymatic activity, to induce a specific functional response or behavior [20,21]. Some of the accounted stimuli are specific to the pathological microenvironments, such as elevated reactive oxygen species and mild acidity in tumors, pH reduction and bacterial enzyme activity in infections, and localized electronegative potentials at bone defect sites, which can serve as effective biochemical triggers for activating bone disease therapies and promoting regeneration [20]. An example is the DDS based on poly(lactic-co-glycolic acid) NPs co-loaded with 17β estradiol (E2) and iron oxide (Fe_3_O_4_), modified with alendronate to achieve bone targeting and realize a magnetically remote-controllable drug release [27].
7. Macromolecular therapeutic-based systems. These systems use water-soluble polymers to direct drugs specifically to bone [10]. Polymers enable selective targeting, extended circulation, enhanced delivery, and controlled cargo release via mechanisms such as physical adsorption, chemical conjugation, and internal loading. Biodegradable, biocompatible, and physicochemically stable polymers are ideal carriers, and biomimetic, bio-inspired systems based on these materials hold significant promises for overcoming current drug delivery challenges [80]. Natural endogenous materials (NEMs), including cells, cell derivatives, polysaccharides, proteins, peptides, and nucleic-acid-based vectors, offer inherent biocompatibility, biodegradability, and natural targeting capabilities, minimizing adverse in vivo reactions and enhancing drug delivery efficacy. Various NEM-based drug delivery systems have been developed to address challenges associated with macromolecules, such as their large size, complex structure, low permeability, and environmental instability [97]. Among polymer-based DDSs, natural polysaccharides are highly favored due to their abundance in nature and renewability [98]. There are even stimuli-responsive polymer-based DDSs that enable selective and targeted therapeutic release at specific tissue sites. Some authors mention examples of pH-, redox-, thermo-, hypoxia-, and enzyme-responsive DDSs [80].
8. Dual-targeting systems. These systems deliver both anabolic and antiresorptive agents simultaneously to different zones of bone, potentially improving therapeutic outcomes without severe adverse effects. Anabolic drugs are favored as they promote the formation of new bone, whereas antiresorptive drugs terminate the further deterioration of bone. The non-specific delivery of anabolic agents results in adverse effects. Several clinical trials have been reported for the combinational therapy of anabolic agents and antiresorptive agents for osteoporosis. However, none of them have proven their cumulative effects in the treatment of disease. Some authors emphasize a dual-targeting drug delivery approach comprising bone anabolic and antiresorptive agents that would simultaneously deliver the therapeutic agents. This approach is believed to intensify the explicit interaction between the therapeutic agent and bone surfaces separately without developing severe adverse effects and to improve osteoporotic therapy effectively compared to non-targeted drug delivery [99].
9. Vesicles derived from macrophages-based systems, as well as other biomimetic carriers. Exosomes, microvesicles, and vesicles originating from reconstructed membranes may retain their chemotactic migration capability and excellent biocompatibility. Exosomes are emerging as novel nanoscale biopharmaceutical DDSs due to their ability to mediate signal exchange through ligands, nucleic acids, or protein factors embedded in their membranes or encapsulated within them [60]. Due to these distinctive characteristics, these systems emerge as promising contenders for innovative drug delivery systems in precision nanomedicine [24]. Biomimetic nanocarriers are developed by incorporating entire cells or cellular components sourced from autologous biological materials. These systems offer excellent biocompatibility and benefit from relatively straightforward fabrication methods, avoiding the need for extensive chemical modification. Derived from natural cellular structures, these bioinspired nanocarriers preserve intrinsic cellular functions. When tailored for specific therapeutic applications, they demonstrate high target specificity, thereby enabling safer and more effective strategies for disease diagnosis and treatment [76]. To encapsulate NPs for drug delivery within a biomimetic outer shell, some authors have mentioned using membranes from white blood cells, red blood cells, platelets, cancer cells, dendritic cells, keratinocytes, VLA-4, or outer membrane vesicles derived from Gram-negative bacteria, as well as hybrid cell platelet–macrophage membranes, hybrid cell erythrocyte–macrophage membranes, and exosomes, which are naturally occurring secreted membrane vesicles found in various biological fluids [76]. Exosomes, like endogenous substances, exhibit excellent biocompatibility; however, their application is limited by complex purification processes and a low yield. Enhancing exosome production remains a critical focus for future research [26]. Extracellular vesicles, consisting of a lipid bilayer with transmembrane proteins and encapsulated cytosolic contents such as mRNAs and miRNAs, function as mediators of intercellular communication both locally and systemically [100]. Bacterial extracellular vesicles (BEVs) are lipid bilayer nanostructures (20–400 nm) secreted via bacterial autolysis and membrane budding, encapsulating bioactive cargo such as endotoxins, peptidoglycans, periplasmic proteins, and nucleic acids [52]. A notable example involves BEVs engineered to overexpress CXCR4 and incorporate ClyA on their surface, enabling the delivery of SOST siRNA as a promising strategy for refractory osteoporosis therapy [47]. However, the intricate architecture of biological systems presents a significant challenge for the large-scale or industrial production of bioinspired DDSs [52].

**Table 3:** Recent DDSs developed for anti-OP treatment or with significant anti-OP potential, categorized based on their structure.

Category	Basic Material	Type of Carrier	Type of Drug	References
Inorganic NPs	Calcium phosphate NPs	HA co-doped with iron oxide	Small non-coding MiR (microRNA)-21/124	[101]
		Zinc-rich amorphous calcium phosphate	-	[102]
		Tetracycline modified and monostearin coated amorphous calcium carbonate platform	Simvastatin	[103]
	Silica NPs	SBA-15/12-tungstophosphoric acid (TPA)-functionalized SBA-15	Alendronate sodium	[104]
		Zinc-based zeolite imidazolium skeleton (ZIF-8) NPs doped with cerium ions and coated with poly(sodium 4-styrenesulfonate)	Alendronate sodium	[105]
		Thiolated mesoporous silica	-	[106]
		Mesoporous silica and Fe_3_O_4_ composite	Baicalein (5,6,7-trihydroxyflavone)	[107]
	Gold NPs	Selenium-gold multi-shell nanocomposites	Icariin	[108]
	Carbon NPs	Carbon nanohorns—calcium phosphates	Ibandronate	[109]
	Bioactive glass	Bioactive glass doped with magnesium–strontium		[110]
Polymer NPs	PLGA NPs	PLGA loaded with secretome	C–X–C chemokine receptor type 4 (CXCR4)	[111]
	PLGA NPs	PLGA modified with alendronate	17β estradiol (E2) and Fe_3_O_4_	[27]
	Bisphosphonate-based coordination polymers (BPCPs)	Benzene 1,4-bis(bisphosphonic acid) (BBPA)—CA, Zn, Mg	5-fluorouracil	[112]
Microparticles/Microspheres	Calcium phosphates	Carbonated apatite microspheres	Antibodies against sclerostin	[113]
Liposomes	Lipid NPs	DLin-MC3-DMA, Zoledronic acid-DSPC, cholesterol, and DMG-PEG-2000	m7G methylated Runx2 mRNA	[114]
	Solid lipid NPs	Compritol^®^ 888 ATO	Raloxifene	[115]
Extracellular vesicles	Exosomes	Exosomes secreted by mesenchymal stem cells (BT-Exo-siShn3)	siRNA of the Shn3 gene	[116]
	Hybrid exosomes	Fused CXCR4+ exosomes with liposomes	Antagomir-188 (inhibiting miR-188 oligonucleotid)	[117]
	Microvesicles	Small osteoblast vesicles		[100]
Hydrogels	Collagen	Dense collagen hydrogels	Sclerostin Antibody	[118]
	Silica hydrogels	Sodium silicate pentahydrate and silicic-acid-based hydrogels	bis-phosphonates	[119]
	Collagen/chitosan/hyaluronic-acid-based hydrogel	Mesoporous silica particles decorated with hydroxyapatite immobilized in collagen/chitosan/hyaluronic-acid-based hydrogel.	Alendronate	[120]
Micelles	Polymers	mPEG_PLGA	Alendronate and astragaloside	[121]
		AL-P(LLA-CL)-PEG-P(LLA-CL)-MY micelles	Myricetin	[122]
		Amphiphilic polymer composed of methoxypolyethylene glycol amine-glutathione-palmitic acid (mPEG-GSHn-PA)	Dexamethasone	[123]
	Capsaicin nano vesicles	Phosphorylated capsaicin (Cap-p) nano vesicles	Curcumin	[124]
Three-Dimensionally Printed Scaffolds	Hydrogel	Three-dimensionally printed triple-crosslinked hydrogel scaffold based on methylpropenylated gelatin (GelMA) and methylpropenylated alginate (AlgMA)	Glycopyrrolate (GA) and epigallocatechin gallate (EGCG)	[125]
Electrospunned nanofibrous membranes	PLGA	PLGA-PLL (Poly (lactic-glycolic acid-L-lysine))	Parathyroid hormone relative peptides	[126]
10. Three-dimensional scaffold-based systems. Drug delivery through scaffolds represents an innovative alternative to traditional pharmaceutical formulations, enabling the controlled spatiotemporal release of therapeutic agents [58]. There are expectations of developing 3D scaffolds that serve as grafts while simultaneously delivering multiple drugs for anesthesia, anti-inflammatory effects, and other therapeutic purposes [65]. Various strategies have been employed to integrate drug delivery functionalities into bone scaffolds, including drug incorporation within the interparticle porosity of bioceramics, loading into polymer matrices, and the surface coating of porous or mesoporous nanoparticles to achieve high drug loading and sustained release [59]. In the case of osteoporotic fractures, convenient local drug delivery approaches are implant coatings, injectable bone cements, and gels [6]. Scaffolds are not only a substitute for the extracellular matrix (ECM) but can also serve as the delivery vehicle for cells and the carrier for growth factors. Scaffolds affect seeded cells, including cell attachment, migration, and proliferation, thus affecting the efficacy of regenerative medicine [8,67]. In addition to the ability of smart scaffolds to interact with cells and induce the desired cell functions for tissue regeneration, scaffolds can also be used to deliver drugs [8]. Smart scaffolds have been designed with the incorporation of bioactive molecules and nanoparticles and the use of tailored modifications of the physical and chemical properties of the scaffolds. Enhancing bone grafts with additional capabilities, such as drug delivery, opens the door to the development of multifunctional implant materials with improved therapeutic potential [66] (Figure 3).

The advancement of controlled drug delivery systems utilizing scaffolds demands a strong multidisciplinary collaboration across key scientific disciplines, including cell and molecular biology, biochemistry, materials science, bioengineering, and clinical research. Integrating expertise from these fields is essential for meeting the necessary criteria for the effective development and manufacturing of drug delivery systems aimed at bone repair, regeneration, and healing [66]. The development of an effective drug-loaded bone scaffold suitable for clinical application involves several critical steps. These include the careful selection of appropriate biomaterials, along with the evaluation and optimization of both the fabrication techniques and drug-loading methodologies to ensure therapeutic efficacy and functional performance [67]. An effective drug delivery scaffold should meet several essential criteria, such as a homogeneous distribution of the drug within the scaffold, the ability to release therapeutic agents at a controlled and predictable rate, a sufficiently low drug binding affinity to ensure stability within the scaffold at physiological temperatures, and the maintenance of physical, chemical, and biological integrity over the required period [58] (Table 4).

Innovative biomimetic scaffolds should be engineered through biofunctionalization strategies to replicate nanoscale topographical and biochemical cues [26]. Several key factors must be taken into account as active drug delivery relies on the drug diffusing through the carrier matrix to reach the surrounding medium. The presence of small, interconnected pores is a crucial requirement for developing an efficient drug delivery system, as these structures can encapsulate pharmaceutical agents and facilitate their gradual release. The size, distribution, and connectivity of pores primarily influence drug release kinetics, with research indicating that lower porosity in bioceramic scaffolds results in a more pronounced initial burst release of drugs [21,65]. Another important consideration is that pore dimensions should be compatible with the size of drug molecules or other bioactive compounds to achieve a controlled, slow-release system. Additionally, for effective bone regeneration, interconnected pores with an average diameter exceeding 100 µm are essential, as they promote bone cell infiltration and proliferation necessary for new tissue formation [66]. The mechanisms underlying adsorption are often influenced by factors such as scaffold composition, surface topography, hydrophobicity, heterogeneity, and surface charge [63], while mechanical properties drive specific interactions with bone cells [21]. The complex physiological environment can affect the effectiveness of nanosized drug delivery, leading to nonspecific retention and premature release during transportation [53]. pH levels can significantly impact the release rates of molecules into the surrounding environment. Further challenges include the cost, stability, efficacy, and safety of the biomolecules themselves. To address these limitations, emerging 3D printing technologies are being developed to integrate a variety of biomolecules into HA scaffolds, potentially overcoming current obstacles [63].

Advanced DDSs for osteoporosis represent a promising research frontier, aiming to enhance drug targeting and bioavailability and minimizing side effects. Innovations involving nanomaterials, hydrogels, liposomes, and 3D printing are key to developing more effective and patient-compliant therapies. Despite advancements, DDSs for osteoporosis face several challenges:

1. Low drug-loading capacity: For example, PLGA nanoparticles, while widely studied, suffer from a low drug-loading capacity, limiting their clinical translation [127]. Efforts to improve drug loading are crucial.
2. Targeting specificity and delivery efficiency: For example, nanocrystals have been found to be superior to other nanoparticle systems as carriers for hydrophobic drug delivery due to their high drug-loading capacity, minimal excipient requirements, chemical stability, and low toxicity. Their nanoscale enhances the bioavailability of poorly water-soluble drugs, making them favorable for industrial application [128]. Improving targeting methods and delivery efficiency remains a challenge.
3. Controlled drug release: Developing systems that offer precise control over drug release to optimize therapeutic outcomes through an autonomous response to lesion-specific pathological cues or external physical stimuli, thus influencing cell behavior and promoting bone repair [20].
4. Biocompatibility and biodegradability: Ensuring that nanomaterials are biocompatible and biodegradable to avoid long-term toxicity. While nanomaterials have shown promising therapeutic effects, caution is needed due to the heterogeneity between animal models and human diseases, as well as variability across disease stages and patient populations [129].
5. Clinical translation: Translating preclinical research to clinical trials requires overcoming hurdles related to biocompatibility, toxicity, and regulatory approval. Regulatory challenges in developing drug delivery need to demonstrate safety and efficacy amid complex drug–carrier–host interactions. Comprehensive preclinical studies and the development of representative animal models are essential. Achieving controlled, sustained drug release while minimizing systemic side effects remains a critical hurdle [130].

Cell therapies offer promising solutions to address the bioacceptability challenges associated with drug delivery systems. However, clinical trials remain essential for validating the efficacy of these advanced delivery approaches. Strategies such as the use of inorganic mesoporous nanoparticles, microfluidic technologies, and molecularly imprinted polymers have also been explored to overcome various limitations in drug delivery [78].

### 4.3. Smart Materials and 4D Printing

Some authors propose four levels of smartness for biomaterials, namely inert (are biocompatible), active (can offer a one-way, uncontrolled release of therapeutics), responsive (can sense specific signals found in the environment or biological processes to then release therapeutics), and autonomous (can sense a signal, release a specific payload, and adapt their properties to changing conditions to keep providing additional, advanced, and/or alternative forms of therapeutics), according to their degree of interaction with the (bio)environment and, specifically, with biological/cellular processes [21]. Smart materials possess the capability to respond dynamically to environmental stimuli, such as changes in temperature or pH [20,131]. In the case of responsive and autonomous materials, the drug release is triggered by environmental stimuli such as temperature, pH change, chemical and redox reactions, and enzymes, or by external stimuli such as electric or magnetic field, light, etc. (Table 5). Some of these smart materials can interact with tissues and cells, promoting cell differentiation, proliferation, and eventually an increase in osseointegration and bone regeneration. Others can be used for the controlled release of drugs, growth factors, or the development of medical devices [21].

Some authors propose a smart biomaterials categorization into two types: those responsive to intrinsic material properties, such as topography, mechanical strength, surface charge, and chemical composition, and those activated by external stimuli, including piezoelectricity, magnetic fields, pH variations, and enzymatic activity. These are further classified into internal microenvironment-responsive (triggered by oxidative species, acidic environment, endogenous electric field, specific ionic concentration, specific enzymes, or specific immune environment) and external stimuli-responsive strategies (Table 6), as well as multi-responsive strategies [20].

Dynamic physiological conditions are an important factor to consider in developing smart materials. These conditions can be exploited to design DDSs that respond specifically to the microenvironment of a target site, such as pH-responsive polymers. Effective therapeutic delivery remains a significant challenge due to numerous physiological barriers, including the necessity to target specific cells and tissues, evade immune system detection, and ensure the precise delivery of therapeutic agents to designated intracellular compartments. This perspective is further complicated by minor variations in the individual patient physiology and disease state that can significantly affect the performance of DDSs, potentially leading to therapeutic failure [132].

pH and redox potential triggers are particularly compelling due to the natural variations in these parameters across different biological environments. After intravenous administration, polymeric nanoparticles are typically internalized by cells via endocytosis, accumulating in late endosomes and lysosomes, where the pH drops to 4.7–5.5 significantly lower than the extracellular pH of 7.4. In addition, the primary therapeutic targets are often located in the nucleus or cytosol, the latter of which is significantly more reducing than the extracellular environment, with intracellular glutathione (GSH) concentrations ranging from 2 μM to 10 mM. As a result, a widely adopted design strategy for smart DDSs leverages these pH or redox differences to trigger targeted nanoparticle activation and therapeutic release [133].

It is possible to create DDSs by carefully selecting enzyme-degradable materials and designing DDSs responsive to physiological conditions. The in vivo environment is complex, and predicting the exact degradation kinetics of DDSs is difficult due to variations in enzyme concentrations and physiological conditions. Polymers like PLGA can be incorporated into DDSs, and their degradation is influenced by esterase enzymes present in vivo [134].

Novel smart scaffold constructs include smart constructs for periodontal regeneration, smart dental resins that respond to pH to protect tooth structures, smart pH-sensitive materials to selectively inhibit acid-producing bacteria, smart resins to modulate biofilm species towards a healthy composition, and the smart tailoring of materials to avoid drug resistance [8].

Poor medication adherence is a major contributor to the discrepancies often observed between results from randomized clinical trials and those obtained in everyday clinical practice. The therapeutic benefits demonstrated by a drug under highly controlled conditions may fail to translate into real-world clinical outcomes if patients do not adhere to prescribed treatment regimens [25]. Three-dimensional-scaffold-based DDSs could bring significant improvement to treatment in this regard. Several anti-OP drugs were already incorporated within targeting drug delivery scaffolds, such as raloxifene, bisphosphonates, BMP, strontium ranelate, parathyroid hormones, and salmon calcitonin [58].

**Figure 4:**
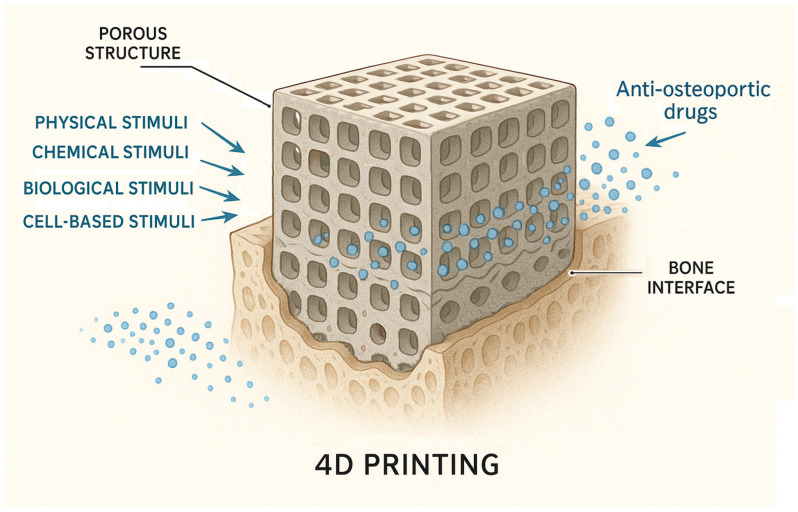
Four-dimensional printing structure combining the benefits of 3D-printed scaffolds with the stimuli-responsive properties of smart materials.

Four-dimensional printing represents an innovative advancement that combines additive manufacturing techniques with stimuli-responsive materials, allowing for the fabrication of complex, multi-scale structures that can alter their shape or function in a predictable and controlled way when exposed to specific external energy sources [53,96,131]. Advanced or ‘smart’ systems can enable targeted drug delivery and leverage the combined action of multiple therapeutic agents to boost osteoblast growth and maturation, further aiding the bone regeneration process [8]. The dynamic nature of 4D-printed constructs arises from their ability to transition between multiple states, typically from a temporary to a permanent configuration, when exposed to external stimuli. Their time-responsive adaptability allows these implants to dynamically respond and adjust to the healing process [72]. Unlike traditional 3D-printed models, which are static and fixed in their initial form, 4D-printed materials can undergo shape transformations in response to external stimuli [131,135]. Some authors indicate the following common shape-shifting abilities of 4D-printed scaffolds: folding (or bending), rolling, torsion, compression, stretching, expansion, shrinkage, and stiffness [136,137]. This adds a layer of complexity to the design process, requiring careful consideration of three key factors: (i) the choice of stimuli-responsive materials, (ii) the types of external stimuli applied, and (iii) the rational design of the construct’s geometry and/or topology [138] (Figure 4).

Structural designs enabling conformational changes typically involve (i) bi- or multilayer configurations that integrate materials with distinct properties to create reversibly deformable structures, and (ii) programmed patterning, which leverages the anisotropy of printed layers to induce differential deformation, such as variations in transverse and longitudinal shrinkage, and other complex directional shape transformations [137].

Compared to 3D printing, 4D printing offers several advantages, including shape transformation and adaptability, the use of smart and responsive materials, self-assembly capabilities with size reduction, enhanced durability and self-healing properties, customizable patient-specific medical applications, and reduced material waste [56]. The adaptive responsiveness of 4D-printed devices to biological signals presents a promising opportunity for developing next-generation medical devices that integrate structural support with dynamic, real-time functionality. Among possible devices built through 4D printing, a wide range of equipment may be included, such as cardiovascular implants, stents, skin coverings, adjustments, implants for orthopedics, frameworks, detectors, and braces [56]. Conventional 3D-printed scaffolds are inherently static and unable to respond to the evolving requirements of bone regeneration. In contrast, 4D printing overcomes this limitation by enabling the fabrication of dynamic scaffolds that can undergo changes in shape, porosity, or mechanical properties over time to enhance bone integration and healing. For instance, a 4D-printed scaffold may initially provide robust mechanical support but gradually degrade in response to tissue formation, thereby facilitating a more natural and effective regenerative process.

In 4D printing, the fabrication process typically employs conventional 3D printing techniques, such as fused deposition modeling (FDM), direct ink writing (DIW), inkjet printing, stereolithography (SLA), or digital light processing (DLP), with slight modifications, most notably temperature adjustments, to enable the printed structure to acquire the necessary form and properties for dynamic movement [135,137,139]. Also, the basic materials employed for 4D printing are vastly used for 3D printing as well, some authors mentioning hydrogels (e.g., gelatin methacryloyl (GelMA)), thermoplastic polymers and their composites (e.g., PLA, PCL), photocuring resins, liquid crystal elastomers, etc. [137].

Three main paradigms define the scope of 4D printing: (i) the use of smart materials capable of undergoing shape transformation upon exposure to external stimuli, enabling the formation of new structures; (ii) the fabrication of polymer-based scaffolds to support cellular and tissue development, particularly in bioprinting applications, requiring biodegradable polymers that degrade in synchrony with tissue maturation; and (iii) the self-assembly of microscale smart particles, which dynamically reconfigure their spatial organization in response to environmental cues [74].

One example of 4D printing research for bone tissue application is the developing of 3D-printed magnetic Fe_3_O_4_ nanoparticles containing mesoporous bioactive glass/polycaprolactone composite scaffolds [140]. This study indicated potential for enhanced osteogenic activity, local anticancer drug delivery, and magnetic hyperthermia. Interesting research is the development of near-infrared (NIR)-responsive double-network shape memory hydrogels that were formed by chemically cross-linking Pluronic F127 diacrylate macromer and the physical blending of PLGA with graphene oxide, an energy converter, to convert NIR irradiation to thermal energy. The hydrogels were manufactured with 3D printing technology using ultraviolet light polymerization [141]. A porous smart nanocomposite scaffold integrating shape memory functionality and controlled growth factor release is composed of chemically cross-linked poly(ε-caprolactone) (c-PCL) and hydroxyapatite nanoparticles, loaded with bone morphogenetic protein-2 (BMP-2). This scaffold demonstrates effective shape memory recovery, transitioning from a compressed state with deformed pores (33 μm in diameter) back to its original porous structure with 160 μm diameter pores [142].

Although still in its developmental phase, 4D printing is regarded as a highly promising innovation due to its potential to significantly improve biomedical processes by enhancing efficiency, reducing costs, and minimizing resource consumption. Nonetheless, the successful implementation and large-scale adoption of this technology will depend on addressing several critical challenges, including the scalability of production, the advancement of suitable material systems, and the assurance of long-term reliability [53]. Some authors also mention that, for the successful integration of 4D bioprinting into clinical applications, four essential criteria must be fulfilled: (i) the selection of an appropriate stimuli-responsive biomaterial, (ii) the use of a safe and effective external stimulus, (iii) the implementation of a rational design strategy to achieve the desired shape transformation, and (iv) the application of a suitable 3D technique [138]. Also, the advancement of 4D printing is constrained by technical limitations of current printing methodologies, the restricted availability of responsive smart materials, and an insufficient understanding of how material properties influence both printability and the functional performance of the final constructs [74].

Despite their potential, smart stimuli-responsive materials face several challenges that hinder clinical translation. Key issues include an insufficient understanding of their immunogenicity, metabolic pathways, and biodistribution. Achieving an appropriate and controlled biodegradation rate is crucial, especially for complex, multi-component biomaterials. Additionally, their application remains limited to specific stimuli modalities. Further progress requires the optimization of operational parameters, the selection of suitable animal models, and in-depth mechanistic investigations [20].

## 5. Discussion

Osteoporosis has emerged as a significant global health concern, presenting substantial clinical, societal, and economic challenges. The increasing life expectancy is a primary contributor to the rising prevalence of this condition, emphasizing the need for heightened awareness among healthcare systems and industry stakeholders. As the aging population expands, the incidence of osteoporosis is expected to rise correspondingly. Consequently, the pharmaceutical sector is likely to maintain a strong interest in the development of effective therapies for this widespread and progressively impactful disorder.

Various strategies, such as optimized crystal growth, solid dispersions, inclusion complexes, and novel preparation methods, have been developed to enhance the bioavailability, stability, and therapeutic efficacy of some anti-OP drugs, addressing challenges related to solubility and patient compliance [32]. However, numerous new possibilities open through the use of DDSs and scaffold-based DDSs.

Inspired by natural systems, the integration of nanomaterials with biomimetic strategies allows these carriers to evade immune surveillance. Although NP-based DDSs offer substantial therapeutic benefits, their exogenous origin often makes them prone to detection and clearance by the host immune system. Through biomimicry, this limitation can be effectively addressed, enhancing circulation time and therapeutic efficacy [76]. When bioinspired, biocompatible DDSs can evade rapid clearance, they may pose risks of mutagenicity, proliferation, and toxicity. Moreover, repeated administration may provoke immune reactions, such as accelerated blood clearance observed with PEGylated nanoparticles, highlighting the need to optimize dosing regimens and determine safe, effective concentration thresholds [52].

Another future perspective is the one offered by the emerging technologies, such as sense-and-respond systems and pulsatile-release platforms, that offer the autonomous regulation of drug levels and replicate multi-dose regimens with a single administration. Bioinformatics and computational modeling represent powerful tools for predicting and optimizing the performance of nano-based DDSs, thereby informing the design process and expediting the development of more effective therapeutic strategies [48]. Regarding scaffold development, computational approaches, although not eliminating the need for the bench-top and preclinical testing of scaffolds, effectively manage interdependencies among design and manufacturing parameters. Furthermore, they enable the early-stage screening of design candidates, thereby reducing the risk of failure during later stages of therapeutic application [62].

Administering bone formation promoters followed by bone resorption inhibitors has been shown to significantly improve BMD at the spine, femoral neck, and total hip. This sequential therapy approach for postmenopausal osteoporosis also results in a greater reduction in fracture risk compared to monotherapy or concurrent combination therapy [143]. In the future, this approach can be transferred to DDS development and 4D printing, greatly expanding the possibilities for adequate treatment of osteoporotic bone defects. Of course, much research is still needed before we can obtain significant results.

The scaffolds serve as a structural framework that supports bone repair and remodeling. Despite their potential for improving bone defect healing, scaffolds present critical limitations, including high production costs, the potential tumorigenicity of incorporated growth factors, and poor integration with native tissue. Additionally, complications such as inflammation-induced implant fractures, loosening, and osteolysis continue to pose significant clinical challenges [20]. Three-dimensional printing technology is essential for the accurate and effective fabrication of biomimetic materials. Recently, scaffolds replicating the 3D porous architecture of native bone have been developed using advanced 3D printing techniques. However, some suggest that multimodal therapies are needed for treating osteoporotic fractures. This complex approach includes MSC transplantation, exosome-based drug delivery, biomimetic materials, and 3D printing. Research exploring this integrated approach still remains limited [60].

When loaded with therapeutic agents, bone scaffolds function as localized DDSs, enabling the site-specific targeting of bone tissue. This localized delivery facilitates adjunctive treatment at the site of injury and contributes to the acceleration of the healing process [67]. This strategy minimizes the need for repeated administrations, allows for the release of one or more active substances at predetermined rates, and protects drugs within the body until their targeted release. Such characteristics contribute to a reduction in drug-related side effects and minimize dosage fluctuations associated with multiple administrations [58]. In DDSs, which rely on small-scale 4D-printed constructs, further microscale studies are needed to demonstrate precise remote control. Additionally, to enable biomedical devices that can reversibly transition between permanent and temporary shapes without energy loss, the long-term sustainability of 4D-printed structures must be further enhanced [138].

While NP-based scaffold–DDS systems hold great promise for the treatment of osteoporosis, their clinical translation is hindered by significant scientific, regulatory, economic, and ethical challenges.

Addressing the scientific issues through innovative design and collaborative research will be key to unlocking their full therapeutic potential. The successful approval of a nano-medicine drug product requires overcoming numerous challenges throughout the development processes. Specialists from pharmaceutical companies are consistently advancing the development and application of novel technologies for the characterization of their specific drug products. Emerging analytical techniques are increasingly employed to address the complexity of nanomedicine formulations [144].

The translation from laboratory to clinical application introduces another layer of complexity. Effective communication and alignment between industry stakeholders and regulatory bodies are essential for the clear interpretation and evaluation of regulatory submissions [144]. Regulatory approval processes, comprehensive safety evaluations, and the need for scalable and reproducible manufacturing methods represent major barriers to clinical adoption. Preclinical studies in humanized mouse models can help to evaluate the interactions of DDSs with the human immune system, bridging the gap between preclinical and clinical applications [145]. A clearly defined framework such as physiologically based pharmacokinetic (PBPK) modeling can be used to predict the in vivo bioequivalence of drug formulations [146], helping to optimize DDS design and reduce the risk of clinical failure. The uniform size distribution, uniformity of dose, charge density, and pharmacokinetic profile of DDSs are essential in these formulations. This approach can facilitate the transition from pre-clinical studies to clinical trials by providing a quantitative understanding of drug behavior.

Regulatory agencies require well-defined manufacturing processes and quality control measures to ensure the consistency and reproducibility of DDSs. Establishing standardized protocols for DDS development and manufacturing is crucial for meeting regulatory requirements and gaining clinical approval. Regulatory pathways for DDSs must consider the unique characteristics of these systems and ensure patient safety and therapeutic benefit [147]. Regulatory challenges are particularly evident in light-responsive 4D printing. Unlike FDM, which can utilize commercially available medical-grade polymers, vat photopolymerization techniques depend on specially synthesized photopolymers. These materials frequently pose greater obstacles in meeting regulatory approval due to their novel and less-characterized nature [148].

Additionally, the high cost of production may restrict widespread use, underlining the importance of developing cost-effective and scalable technologies [46]. It is estimated that experimental platforms for the drug delivery of new bone treatment will need a large amount of time to transform before being used in clinical settings [59]. Multicompartment systems are also associated with several challenges, such as formulation complexity, variability in raw material properties, stability concerns, and limitations in scalability during manufacturing processes [80]. Ensuring a reliable and consistent supply of high-quality materials is crucial for the scalable production of DDSs.

Biocompatibility is a key requirement for biomedical applications of 4D printing. Several studies have focused on evaluating the biocompatibility of materials used in 4D printing (e.g., hydrogels, shape memory polymers) [149,150]. Many 4D printing applications utilize biodegradable polymers that can be broken down by the body’s natural processes. Poly(vinyl alcohol) (PVA) is one such polymer gaining traction in 3D and 4D printing due to its versatility and applicability in medical and pharmaceutical fields [151]. Some authors indicate a good biodegradability of 4D constructs based on a light-active composite through incorporating gold nanoparticles into a shape memory polyurethane matrix [152]. The substantial molecular weights and extended chain lengths of pH-responsive polymers make them well suited for oral and transdermal drug delivery; however, their use in parenteral applications is restricted due to potential toxic interactions and unpredictable biodegradation profiles [132]. Despite the advancements, several challenges remain in ensuring the long-term biocompatibility and predictable degradation of 4D-printed materials: the standardization of biocompatibility testing in vitro and in vivo, especially for long-term in vivo studies, assuring a controlled and predictable degradation, and the continued development of novel materials.

This review explored various strategies for managing osteoporosis, emphasizing the use of scaffolds for targeted drug delivery to bone tissue. Scaffolds offer significant benefits, including the stimulation of osteoblast activity and the inhibition of osteoclast-mediated bone resorption, while targeted delivery minimizes the adverse effects associated with systemic therapies. Collectively, these advanced solutions aim to enhance therapeutic efficacy, minimize adverse effects, and enable targeted and personalized treatment strategies. Nevertheless, further research is essential for optimizing treatment approaches for bone-compromising conditions such as OP. We hope that the findings presented in this paper will contribute to advancing research on OP and support a more effective evaluation of DDS and scaffold properties.

## 6. Conclusions

Research is ongoing in the field of bone tissue engineering, especially for the management of difficult-to-heal fractures, like the ones in osteoporotic patients. OP is a chronic metabolic disease characterized by high prevalence and serious complications. Multifunctional DDSs based on 3D- and 4D-printed scaffolds, with enhanced osteogenic properties, hold significant potential for integration into biomedical clinical practice, owing to their multiple therapeutic advantages. This advancement aligns with the growing trend toward personalized and precision medicine, where therapies are tailored to individual patient needs.

## Figures and Tables

**Figure 3:**
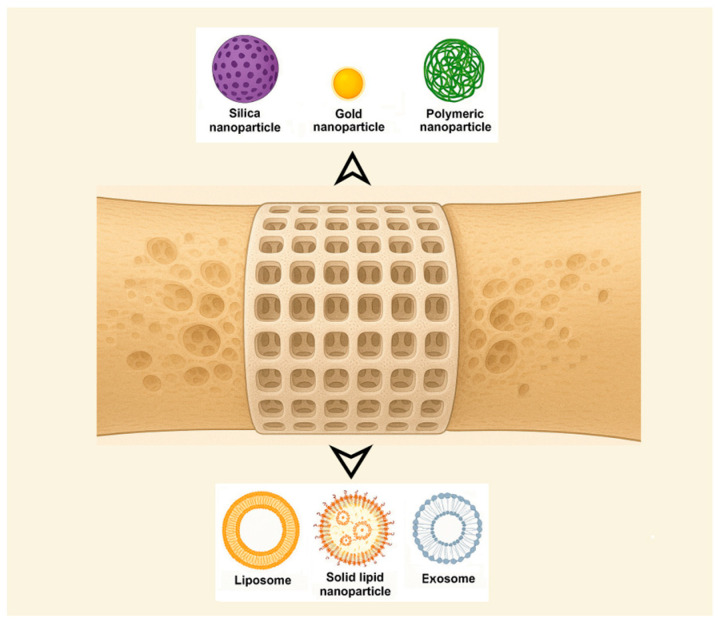
Multifunctional theoretical 3D-printed scaffolds integrating different possible DDSs for bone repair and regeneration.

**Table 1:** Classification of drugs used in OP treatment [17,50,51].

Classification	Drug Type	Representative Drugs	Adverse Reactions
Bone-resorption-inhibiting drugs	Bisphosphonates	Alendronate sodium	Skeletal muscle pain
	Calcitonin	Salmon calcitonin	Allergic reactions, hypocalcemia
	Estrogenic	Raloxifene	Thrombocytopenia, gastrointestinal reactions
	Monoclonal antibodies	Denosumab	Cardiovascular injuries
Bone-formation-promoting drugs	Parathyroid hormone-like compounds	Teriparatide	Orthostatic hypotension, tingling sensation in the extremities
	Fluoride	Disodium fluorophosphate	High concentrations are likely to cause bone cancer and neuroarthritis
	Statins	Simvastatin	Liver injury
	Androgen	Testosterone	Cardiovascular and cerebrovascular injuries
	Strontium salt	Strontium ranelate	Myocardial infarction, thromboembolism, DRESS syndrome
Bone nutrition drugs	Calcium	Calcium carbonate	Constipation, hypercalcemia
	Vitamin D and its derivatives	Vitamin D3/cholecalciferol	Hypercalcemia
	Vitamin K	Osteotriol/calcitriol	Hepatotoxicity, hemolytic anemia

**Table 2:** Comparison between most commonly used 3D techniques for orthopedic applications [68,70,71,72,73].

**3D Printing Category**	**3D Printing Techniques**	**Advantages**	**Disadvantages**	**Common Applications**
Extrusion-based 3D Printing	Fused deposition modeling	More compatible materials; Adjustable pore size; Convenience and low cost	Low resolution; Increased material shear due to the extrusion process; Low efficiency	Surgical simulation model
	Bioprinting	Maintaining of biological activity; Precise control of the porous structure; Multimaterial printing	Lower mechanical strength; Low efficiency; Low precision; Limited materials; Acidic degradation products	Orthopedic tissue engineering
Powder Bed Fusion	Selective laser sintering	Powdered materials; High-resolution data; High material utilization	High temperatures affect the biological activity of materials; High cost; Rough surface	Implants
	Electron beam melting	Manufacturing efficiency; High-vacuum environment	Cost and complexity; Material limitations; Rough surface; Low accuracy	Implants
Material Jetting	Inkjet printing	High resolution; Multi-material simultaneous printing; Low cost	Higher material viscosity required; Lower stability	Orthopedic tissue engineering
Vat Photopolymerization	Stereolithography	High accuracy; High precision; Good surface quality	Low efficiency; Limited materials	Surgical guide plate and guide
	Digital light processing	Rapid generation; High accuracy; High precision; Good surface quality	Limited materials	Surgical guide plate and guide

**Table 4:** Key characteristics that an ideal drug-loaded bone scaffold should exhibit [67].

Physical Features	Drug Delivery Requirements	Mechanical Properties	Biological Aspects
Adequate pore size	Suitable drug release profile	Mechanical compatibility	Biofunctional
High porosity	Drug decomposition protector	Mechanical integrity	Biocompatible
High pore interconnectivity		Mechanical strength	Biodegradable
Wettability			Osteoinductive
			Osteoconductive
			Osteogenic
			Osteointegration

**Table 5:** Stimuli in 4D printing are of four distinct types: physical, chemical, biological, and cell-based [20,53].

Physical	Chemical	Biological	Cell-Based
Heat	Solvents	Enzymes	Cell contractile forces
Light	Moisture	Glucose	Cell proliferation
Electric field	pH	Oligonucleotides	
Magnetic field	Ions		
Ultrasound	Redox		
Microwave			
Mechanical forces			

**Table 6:** Comparative table: external stimuli-responsive strategies for bone therapy and regeneration [20].

Stimulus Type	Mechanism of Action	Biological Effects
Photoresponsive	Infrared laser induces photophysical effects; modulates mitochondrial ATP synthesis	Enhances cellular metabolism; promotes angiogenesis and bone regeneration
Magnetic Field	Fe_3_O_4_ NPs generate localized heat under magnetic fields (magnetic hyperthermia)	Improves osteogenic differentiation; may also assist in tumor ablation in bone cancer contexts
Ultrasound-Responsive	Enhances VEGF-A mRNA expression and chondrocyte proliferation	Accelerates bone matrix maturation; stimulates mineralization
Electroresponsive	Electrical currents stimulate BMSCs and upregulate BMPs and cytokines	Promotes BMSC proliferation/differentiation; boosts bone formation through calcium–calmodulin signaling
Piezoelectricity	Mechanical stress induces electric charges in scaffolds or bone tissue	Supports stem cell proliferation and osteogenic differentiation
Mechanical Stimuli	Mechanical strain activates PI3K/Akt and other pathways	Promotes early osteogenic differentiation of stem cells; improves regeneration in osteoporotic bone

## Data Availability

Not applicable to this article.
